# Annotations

**Published:** 1889-12-21

**Authors:** 


### Annotations.

**Mr. Speaker on "Humour and Fun."**

The Mayor of Leamington last week entertained the Speaker of the House of Commons and a distinguished company at the Town Hall of that pleasant Midland resort. In the course of an interesting address, the Speaker said that if, in the House of Commons, "they could substitute for some of the fiery and angry invective they heard some humour and fun in its real sense, they would be a better legislature than they were." There the Speaker spoke like a philosopher and a physiologist. Anger alters the blood-vascular condition of the brain, and makes it physically impossible for the angry person to use the best judgment and reason that are in him. Humour and fun, "in their real sense," as the Speaker put it, dispose to breadth of view, to good sense, to kindly feeling, and so to wisdom and justice. Is there any undeniable occasion for the exceedingly angry feelings which are often displayed in Her Majesty's House of Commons? We are never so dreadfully ill-governed but that our objections may be set forth at least with patience; nor are our statesmen in authority ever so absolutely perfect but that they may listen to criticism with a certain appreciative meekness. Besides, in our humble opinion, a man who speaks angrily, at least, in most cases, goes far to destroy his own cause. The art of politics is eminently a business of expediency. It is not a trifling affair by any means. It should never be committed to fools. On the contrary, it demands the contant exercise of the noblest qualities of the noblest minds. If a man have little nobility of character naturally, he is likely soon to have less if he becomes part and parcel of the House of Commons. On the other hand, if he be really noble in mind and character, there is no better field for the display of his magnanimity and enduring greatness. In any event, anger is a bad and injurious element to introduce, whilst humour and fun are like genial sunshine on a dismal December day. It is quite conceivable that at a great crisis a little wholesome humour, and the forbearance and illumination born of it, might save the State from an overwhelming catastrophe. Few things are more conducive both to public and private health than "humour and fun in the real sense."

**Horses and Asphalte.**

The Horse-Accident Prevention Society—significant name—approached the City authorities last week, with the view of gradually superseding the asphalte of the City streets by wood paving. If the "Horse-Accident Prevention Society" does not succeed in this object it is in contemplation to form a "Man-and-Woman-Accident Prevention Society," in the hope that the statistics of human brain concussions and broken limbs may be more successful than the pitiable stories of the sorrows and sufferings of the horse. We have on several occasions approached from a humanitarian point of view, as becomes our philanthropic *role*, the subject which the Horse Accident Prevention Society approaches from a financial and economic standpoint. The British people are at least humane; and if a mere "Hey, Presto," could evolve a wooden paving from the asphalte without trouble and without cost, no doubt even the City authorities would join in a conjuring chorus. But the prospect of every street in the City "up" for repairing, acts, as Carlyle says, as the rattling of "a bag of dried peas" does upon a flock of turkeys, and "appals the heart of the boldest." We must, however, not take counsel of our cowardice and love of ease in a case of this kind. The accidents to horses within the narrow area of the City, and on its asphalted roadways, are grievous and even shameful; they are, besides, innumerable. How often do the more tender-hearted of City men turn away their heads in despair from the sight of fallen, struggling, and terrified horses, as they pass through the greasy and slippery streets! Sometimes, after a thaw or drizzling rain hundreds of horses will fall and be seriously injured in one day. Look at it as we will the thing is a disgrace to us. We should all be ashamed and disgusted if Spanish bullfights were to come into vogue in England. There is probably more torture inflicted upon horses in a single slippery day in the streets of London city than is endured by all the fighting bulls and horses in Spain in a whole year. Of course we can pharisaically "pooh-pooh" a statement of this kind. But "pooh-pooh" never altered a hard fact, and never will. So long as John Bull makes no effort to save his faithful and intelligent friend the horse from the dread and terror of the City streets, so long is John Bull, from the practical philanthropic point of view, a hardened and lazy hypocrite.

**Mr. Browning and Vivisection.**

It is one of our standing contentions in this journal that leaders of thought and action in one department of human affairs know too little of and pay too little heed to the best judgment and feeling of leaders in other departments. The scientist despises the ranging flights of the poet because he is a poet, and the poet distrusts the humanity of the scientist because he is a scientist. Vivisection is eminently one of those questions which demand for their solution, not the wilful dicta of one or a few able men whose minds work always in one particular groove, but the calm and candid judgment of competent persons who represent all the varieties of activity of which the mind of man is capable. The experimental physiologist insists that he and he alone is able to understand the subject in all its bearings, and that, therefore, persons who know nothing of physiology are out of place in taking part in the discussion. But although the present writer is perfectly familiar with experimental physiology and all its ways, he feels that the question is one which not only permits but demands the larger judgment of cultured and conscientious men of all classes. Mr. Browning, whom all cultured and all honest men mourn at this moment, was known to all the world, not as a maker of *vers de société*, but as a poet of the profounder sort, as a philosopher, as a leader, at least, of some. In one of his latest poems, *Arcades Ambo*, he shows pretty plainly how vivisection strikes him:

" *A.*—You blame me that I ran away?
Why, sir, the enemy advanced:
Balls flew about, and—who can say
But one, if I stood firm, had glanced
In my direction? Cowardice?
I only know we don't live twice,
Therefore—shun death, is my advice.

*B.*—Shun death at all risks? Well, at some
True, I myself, sir, though I scold
The cowardly, by no means come
Under reproof as overbold
—I, who would have no end of brutes
Cut up alive to guess what suits
My case, and saves my toe from shoots."

There is a loud ring of contempt in these lines for the vivisector. The vivisector may, of course, try to indulge his *tu quoque*. But that is entirely unworthy of the occasion. Men of real mental capacity and conscience do not stoop to bandying words. Browning was a great man, with a wide and deep power of vision. He was, too, when he wrote thus, an old man with a lifelong experience. He was a man of culture. Most of our active vivisectors are young men who have fame still to win, and who hope to win it by physiological discovery. In this country the vivisectors who have passed middle life are comparatively few. In Germany and France no doubt the case is different. But Great Britain has seldom taken her cue from any continental nation without regretting it. Our own homely common-sense and simplicity of conscience have always been our best guides. We ought to consider whether a more careful and thorough use of clinical methods will not really advance the healing art more than vivisection. The only conceivable justification of vivisection is the possible knowledge and power it may give to practical medicine. In the long run the mass of mankind, and not the few, determine the merits of every age and of every worker in it. The place of vivisectors will be fixed by the verdict of posterity. No man, who is not a fool, lightly esteems the permanent judgment of mankind at large. It will be well for vivisectors to look at their work from this point of view.

